# Supplementary material for: γ effects identify preferentially populated rotamers of CH2F groups: side-chain conformations of fluorinated valine analogues in a protein
Source: Magn Reson (Gott). 2025 Nov 17;6(2):257–72. doi: 10.5194/mr-6-257-2025 (PMC12753139; doi:10.5194/mr-6-257-2025)
Supplement: The supplement related to this article is available online at https://doi.org/10.5194/mr-6-257-2025-supplement. [file mr-6-257-2025-supplement.pdf]

Supplement of Magn. Reson., 6, 257–272, 2025
https://doi.org/10.5194/mr-6-257-2025-supplement
© Author(s) 2025. CC BY 4.0 License.

Open Access
MAGNETIC RESONANCE

Supplement of

γ effects identify preferentially populated rotamers of CH_2F groups: side-chain conformations of fluorinated valine analogues in a protein

Elwy H. Abdelkader et al.

Correspondence to: Gottfried Otting (gottfried.otting@anu.edu.au)

The copyright of individual parts of the supplement might differ from the article licence.

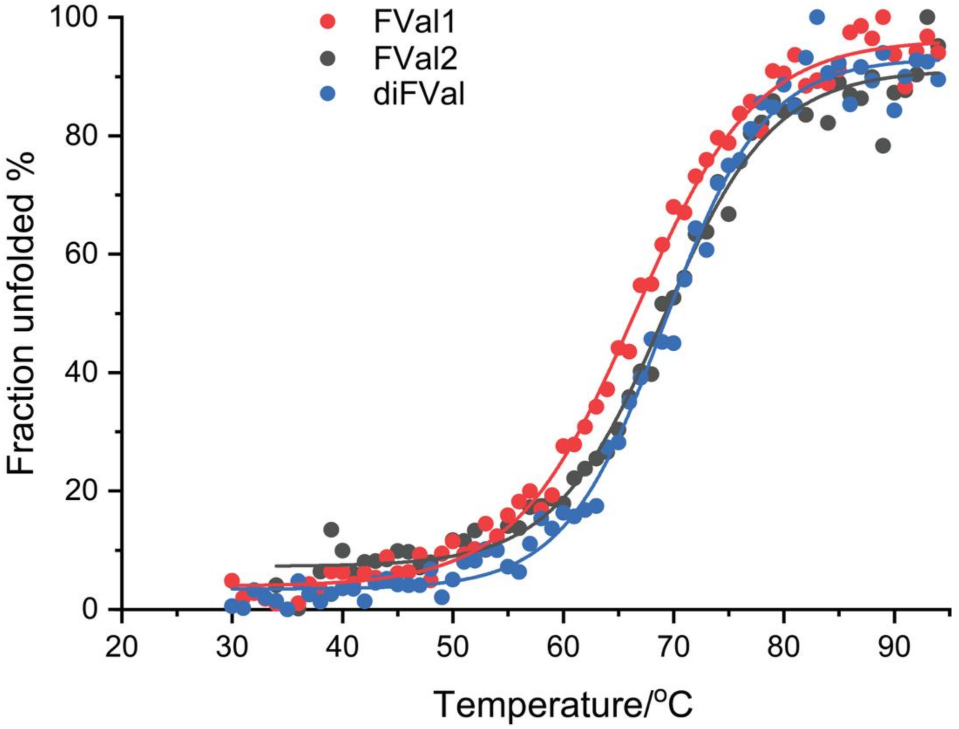

Figure S1. Thermal stability analysis by circular dichroism (CD) spectroscopy. The CD signal of GB1 with FVal1 (red), FVal2 (green) and diFVal (blue) was monitored at 216 nm in a 1 mm quartz cuvette using a Chirascan spectrometer (Applied Photophysics). Parameters used: protein concentration about 0.3 mg mL^{-1}, pH 7.5, heating rate 1 °C min^{-1}. Blanks with buffer measured in the same cuvette were subtracted from the data. For visual clarity, the data were scaled by setting the lowest and highest points measured to 0% and 100%, respectively. The melting temperatures obtained from the fits are 66 °C for GB1 made with FVal1 (GB1-1, red), and 69 °C for GB1 made with FVal2 (GB1-2, black) or diFVal (GB1-d, blue). The melting temperature of the wild-type protein is about 79 °C (Tan et al., 2025).

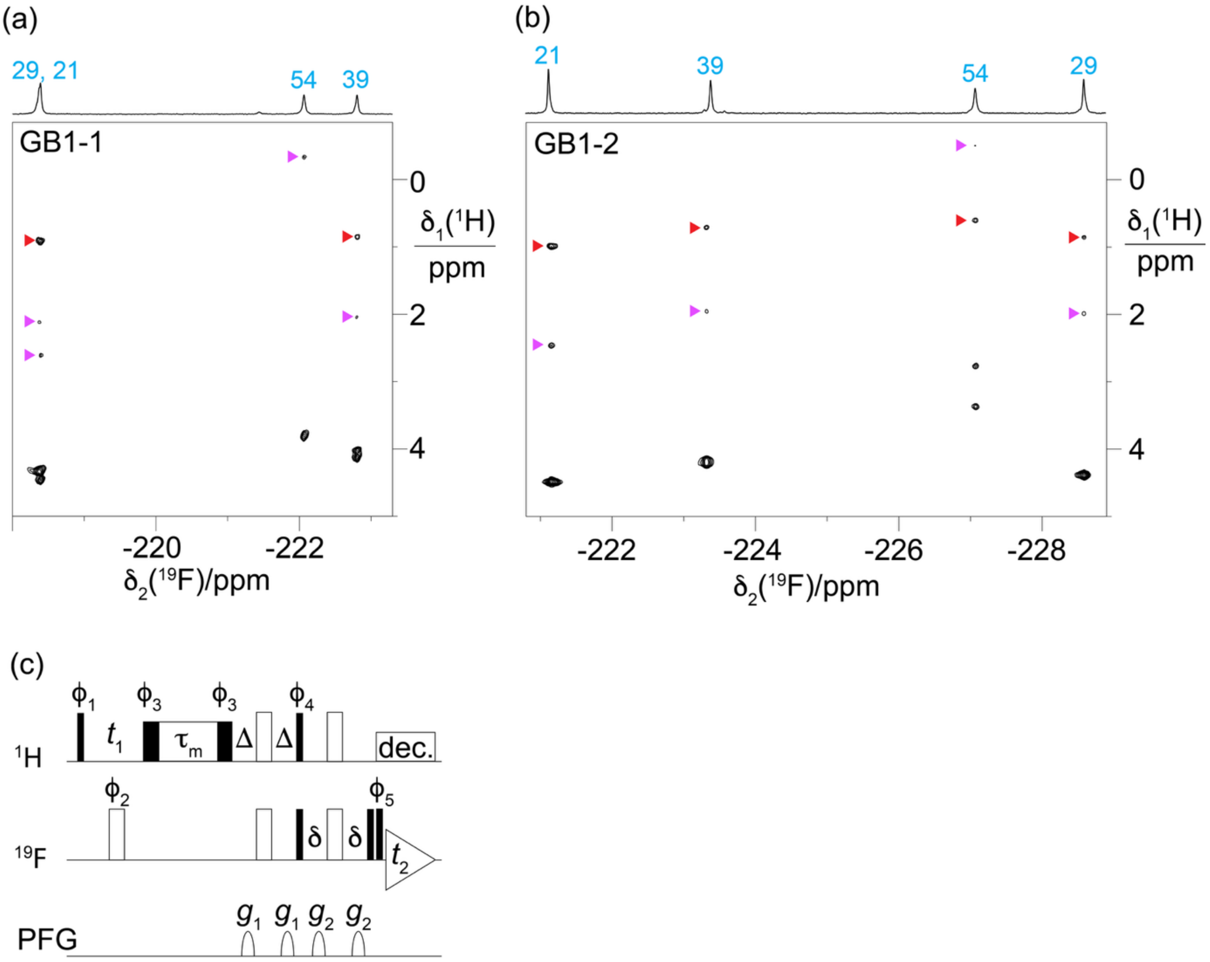

Figure S2. ^{19}F-detected [^{1}H,^{1}H]-TOCSY spectra of GB1-1 and GB1-2. The ^{1}H chemical shifts of H^{β} and $C^{\gamma}H_3$ groups are indicated by purple and red arrows, respectively. The 1D ^{19}F-NMR spectra are shown at the top together with the resonance assignments. (a) Spectrum of GB1-1, recorded in 9.5 h with a mixing time of 70 ms. The ^{1}H chemical shifts of H^{β} and $C^{\gamma}H_3$ groups are indicated by purple and red arrows, respectively. (c) Pulse sequence used to record the ^{19}F-detected [^{1}H,^{1}H]-TOCSY spectra. Narrow filled and wide unfilled rectangles indicate 90° and 180° pulses, respectively. Wide filled rectangles represent 0.5 ms trim pulses. τ_m denotes MLEV-17 mixing (Griesinger1988). The phases of pulses outside the mixing period τ_m are x unless indicated otherwise. Phase cycle: ϕ_1 = y, -y; ϕ_2 = 8(x), 8(-x); ϕ_3 = 2(x), 2(-x); ϕ_3 = 2(x), 2(-x); ϕ_4 = 16(x), 16(-x); ϕ_5 = 4(x), 4(-x); $\phi_{receiver}$ = 8(x, -x), 8(-x, x). Δ was set to 5.2 ms and δ to $\Delta/2$. Pulsed field gradients were sine-shaped and of 1 ms duration, with g_1 = 40 G/cm and g_2 = 13.5 G/cm. ^{1}H decoupling used WALTZ-64.

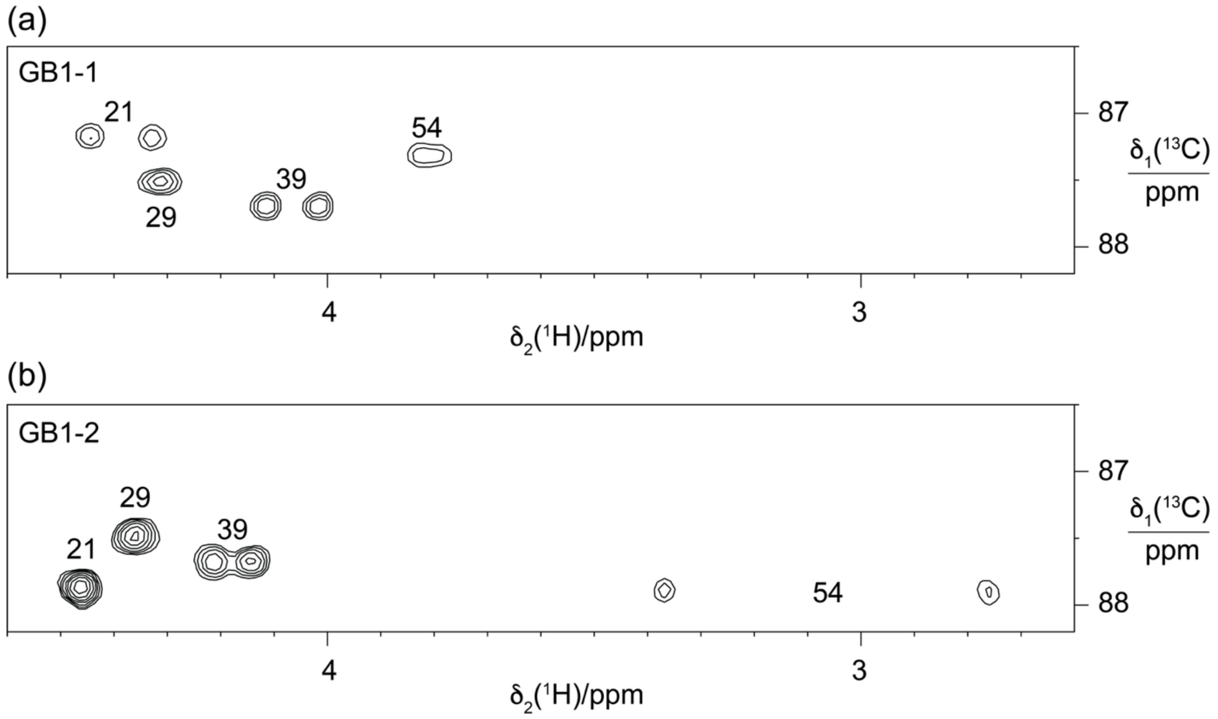

Figure S3. Selected spectral region from ^{13}C-HSQC spectra of GB1-1 and GB1-2 showing the cross-peaks between the protons and carbons of the CH_2F groups. The spectra were recorded with ^{19}F-decoupling using a Bruker 500 MHz NMR spectrometer equipped with a ^{1}H/^{19}F/^{13}C cryoprobe. The spectra were recorded with a spectral width of 109 ppm in the ^{13}C-dimension, revealing that published ^{13}C chemical shifts of the CH_2F groups of FVal2 in the protein PpiB had been reported too low by 30 ppm due to aliasing (Frkic et al., 2024). (a) Spectrum of GB1-1. Parameters used: t_{1max} = 27 ms, t_{2max} = 82 ms, total recording time 3.6 h. (b) Spectrum of GB1-2. Parameters used: t_{1max} = 37 ms, t_{2max} = 82 ms, total recording time 4.1 h.

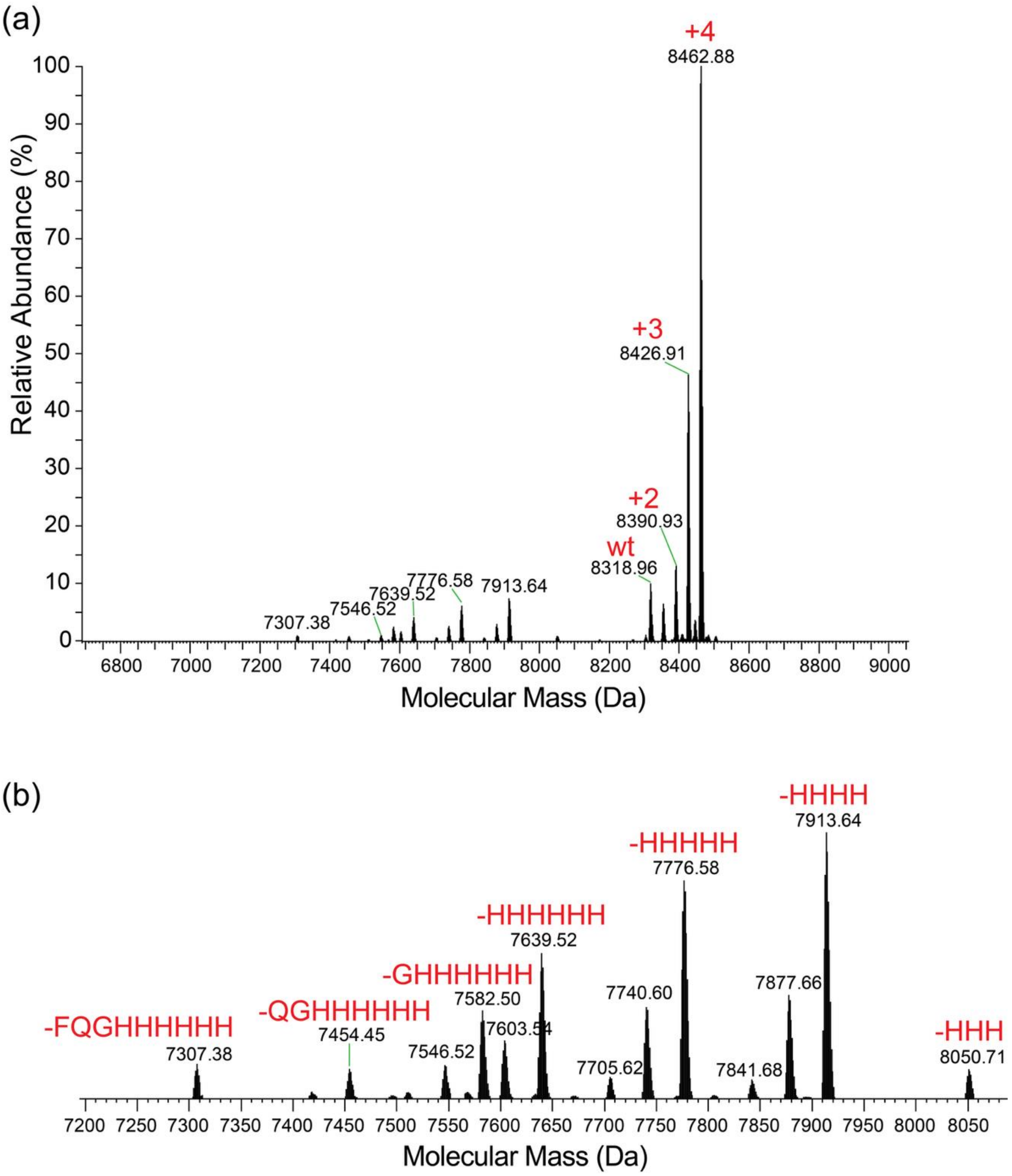

Figure S4. Intact protein mass spectrometry of GB1-d following storage -20 °C for two years. (a) Overview. The expected mass of the full-length protein with four diFVal residues is 8,462.08 Da (8426.08 Da with three diFVal residues and one Val, 8390.08 Da with two diFVal and two Val residues, 8354.08 Da with one diFVal and three Val residues, 8318.08 Da for the wild-type protein). The mass spectrum of the fresh protein was reported by Maleckis et al. (2022). (b) Zoom into the region between 7200 and 8100 Da. The calculated masses of the C-terminally digested protein containing four diFVal residues are, respectively, 8050.59, 7913.45, 7776.3, 7639.16, 7582.11, 7453.98 and 7306.8 Da for the peaks annotated in red.

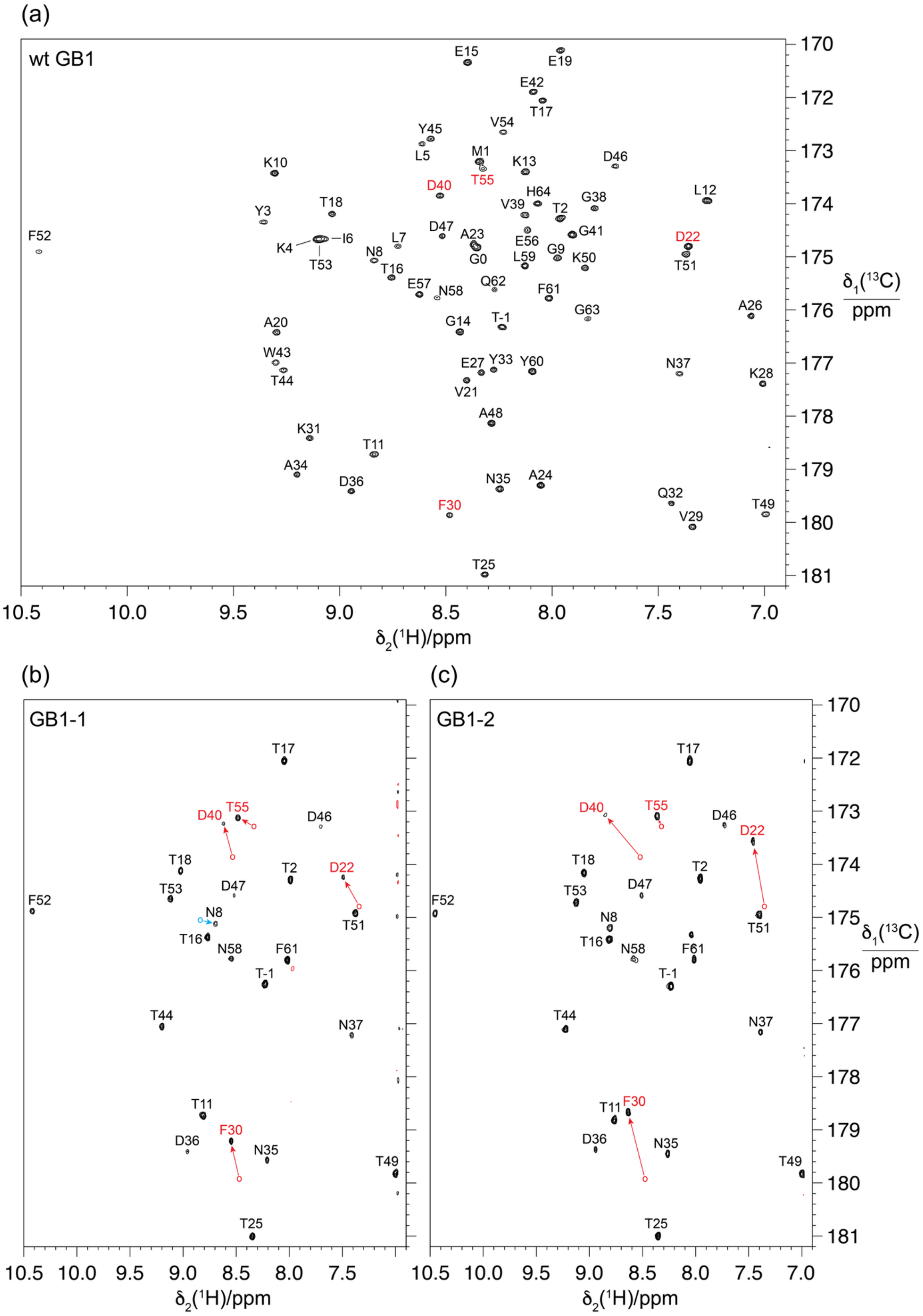

Figure S5. 2D HNCO spectra showing changes in the chemical shifts of the carbonyl groups of FVal1 and FVal2 residues in GB1. The spectra were recorded as the first ^{13}C-^{1}H plane of the 3D HNCO experiment. Parameters: 600 MHz NMR spectrometer equipped with a cryoprobe,

t_{1max} = 42 ms, t_{2max} = 122 ms, total recording time 58 h per spectrum. The cross-peaks are labelled with the assignment of the backbone amide protons, highlighting the residues following valine in red. (a) 2D HNCO spectrum of uniformly ^{15}N-labelled wild-type GB1. (b) 2D HNCO spectrum of GB1-1. To simplify the NMR spectrum, the sample was produced with ^{15}N-labelled Asn, Asp, Phe and Thr. The FVal1 amino acid used contained a ^{13}C label in the CH_2F group (Maleckis et al., 2022). Red arrows indicate the changes in chemical shift of the cross-peaks of residues following FVal1 from the corresponding position in the wild-type protein (indicated by a circle). Among the other cross-peaks, N8 shows the largest shift change (highlighted with a blue arrow). (c) Same as (b), but for GB1-2.

(a)

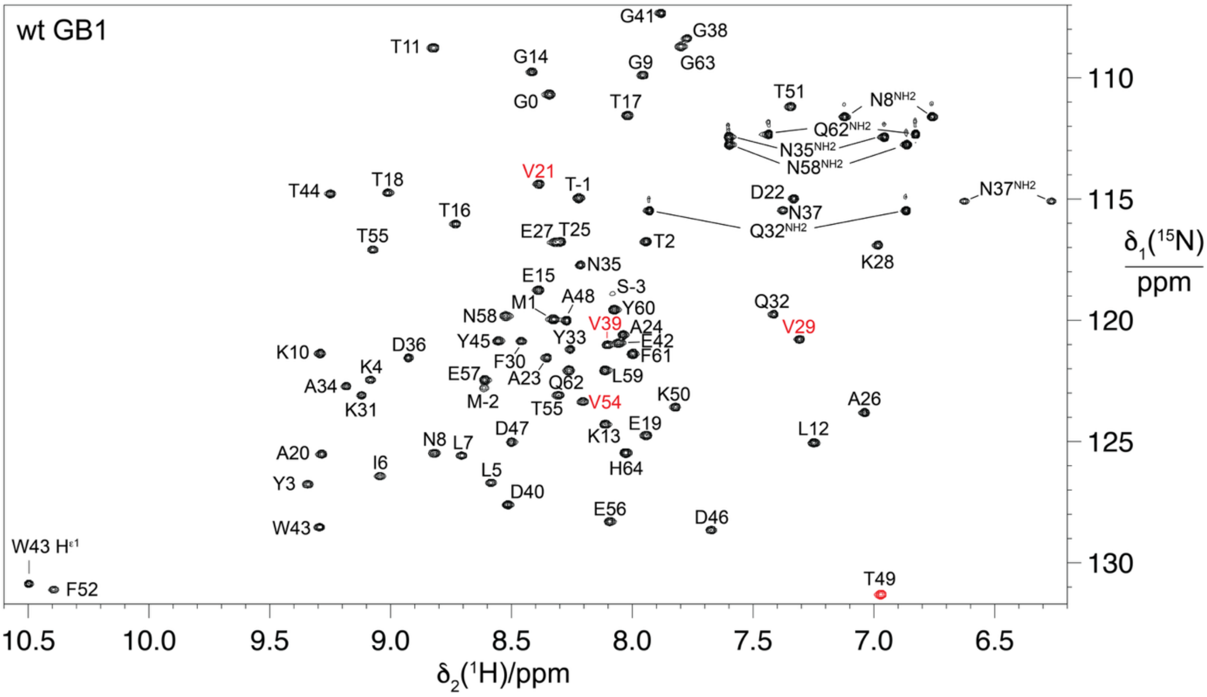

wt GB1
G41
G38
T11
G14
G9
G63
G0
T17
T51
N8NH2
Q62NH2
N35NH2
N58NH2
V21
T-1
T18
T44
D22
N37
N37NH2
T16
Q32NH2
E27
T25
T2
T55
K28
N35
E15
S-3
M1
A48
Y60
Q32
N58
V39
A24
V29
Y45
Y33
E42
K10
D36
F30
F61
K4
E57
A23
Q62
L59
A34
K50
M-2
T55
V54
K31
A26
D47
E19
K13
L12
N8
L7
A20
I6
L5
H64
Y3
D40
E56
D46
W43
W43 Hε1
F52
T49
110
115
120
125
130
δ1(15N) ppm
10.5
10.0
9.5
9.0
8.5
8.0
7.5
7.0
6.5
δ2(1H)/ppm

(b)

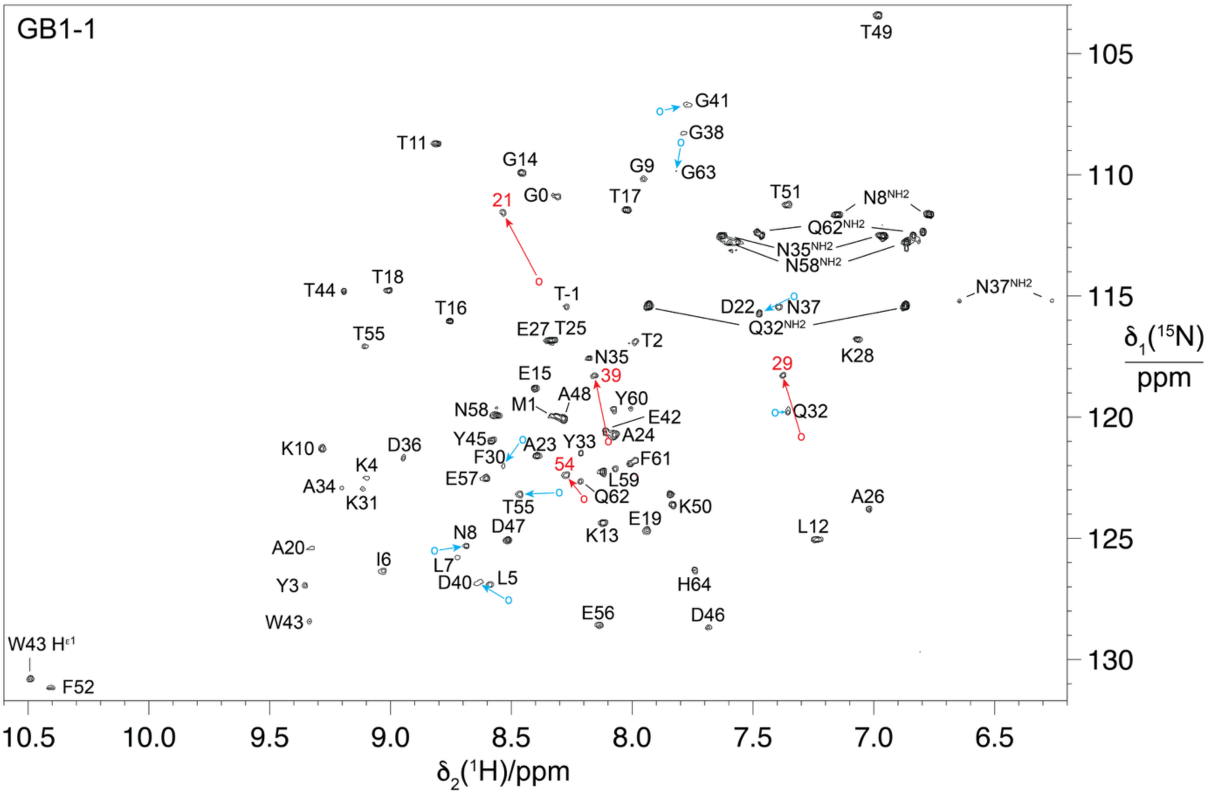

GB1-1
T49
G41
G38
T11
G14
G9
G63
21
G0
T17
T51
N8NH2
Q62NH2
N35NH2
N58NH2
T-1
T18
T44
D22
N37
N37NH2
T16
Q32NH2
E27
T25
T2
T55
K28
N35
29
E15
39
A48
Y60
M1
N58
Q32
E42
Y45
A23
Y33
A24
K10
D36
F30
F61
K4
54
E57
L59
A34
Q62
K31
T55
K50
A26
D47
E19
L12
N8
K13
A20
I6
L7
D40
L5
Y3
H64
E56
D46
W43
W43 Hε1
F52
105
110
115
120
125
130
δ1(15N) ppm
10.5
10.0
9.5
9.0
8.5
8.0
7.5
7.0
6.5
δ2(1H)/ppm

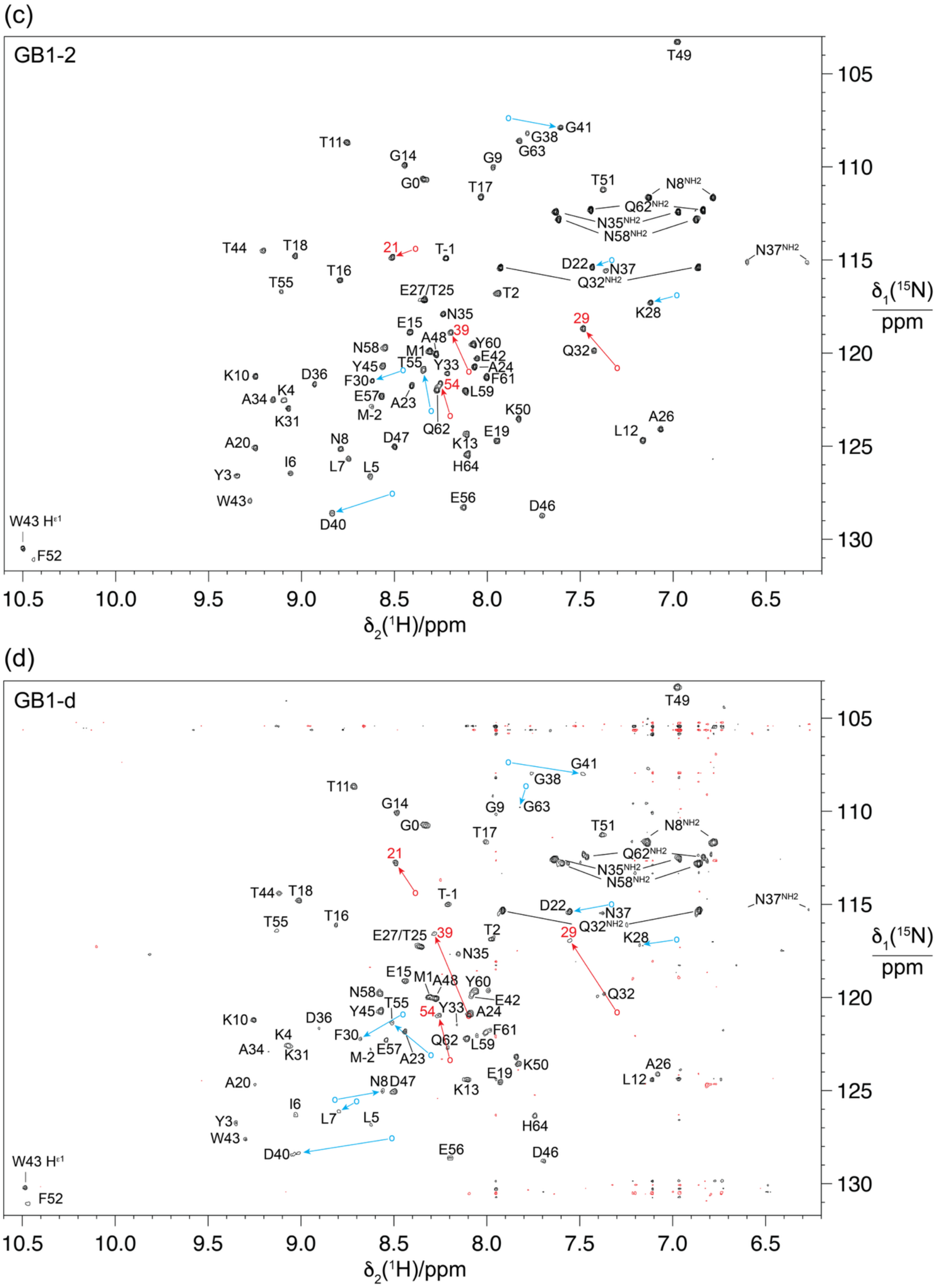

Figure S6. ^{15}N-HSQC spectra of GB1 (wild-type and constructs with valine substituted for fluorinated valines) recorded using an 800 MHz NMR spectrometer equipped with a cryoprobe. The cross-peaks are labelled with the resonance assignments. (a) Spectrum of uniformly ^{15}N-labelled wild-type GB1. The total recording time was 15 minutes. The red cross-peak of T49

is folded. (b) Spectrum of GB1-1 at natural isotopic abundance. Parameters: t_{1max} = 32 ms, t_{2max} = 96 ms, total recording time 102 h. The cross-peaks of the fluorinated valine residues are labelled with the residue number in red. To visualize the changes in chemical shifts, circles mark the positions of the respective cross-peaks in the wild-type protein and arrows indicate the direction of the shift changes caused by the fluorine atoms. Blue circles and arrows identify the most strongly shifted peaks from residues other than valine or fluorinated valine. (c) Spectrum of GB1-2 at natural isotopic abundance. Parameters: t_{1max} = 32 ms, t_{2max} = 98 ms, total recording time 48 h. Same peak labelling as in (b). (d) Spectrum of GB1-d recorded at natural isotopic abundance. Parameters: t_{1max} = 32 ms, t_{2max} = 98 ms, total recording time 15 h. Same peak labelling as in (b).

Table S1. DNA and corresponding amino acid sequence of the GB1 construct used in the current work.

Protein	DNA sequence	Amino acid sequence[a]
GB1	ATGGCTTCTATGACCGGTATGACCTACAAACTGATC<br>CTGAACGGTAAAACCCTGAAAGGTGAAACCACCACC<br>GAAGCGGTTGACGCGGCGACCGCGGAAAAAGTTTTC<br>AAACAGTACGCGAACGACAACGGTGTTGACGGTGAA<br>TGGACCTACGACGACGCGACCAAAACCTTCACCGTT<br>ACCGAAGAAAACCTGTATTTTCAGGGCCATCATCAT<br>CACCATCAC	MASMTGMTYKLILNGKTLKG<br>ETTTEAVDAATAEKVFKQYA<br>NDNGVDGEWTYDDATKTFTV<br>TEENLYFQGHHHHHH

[a] The present work uses the common sequence numbering of wild-type GB1 (Juszewski et al., 1999). The N-terminal MASMTG tag was numbered -5 to 0. The N-terminal methionine was lost during protein expression.

Table S2. T_1(^{19}F), R_2(^{19}F) and $R_{1\rho}$(^{19}F) values of GB1-d.[a]

Sequence position and stereospecific assignment of the CH_2F group	T_1(^{19}F)/s	R_2(^{19}F)/s^{-1} (uncertainties from fitting in brackets)	$R_{1\rho}$(^{19}F)/s^{-1} (uncertainties from fitting in brackets)
29, γ_1	0.43	15.9 (0.1)	14.5 (0.3)
21, γ_2	0.40	9.8 (0.2)	9.3 (0.5)
54, γ_1	0.58	26 (1)	23 (2)
21, γ_1	0.58	11.6 (0.4)	12.1 (0.5)
39, γ_2	0.36	19 (1)	16.8 (0.5)
39, γ_1	0.36	21 (2)	18 (0.6)
29, γ_2	0.42	15.6 (0.5)	13.9 (0.4)
54, γ_2	0.61	36 (1)	26 (1)

[a] T_1(^{19}F) values were determined from the zero crossings in inversion recovery experiments with an estimated precision of ±0.02 s. R_2(^{19}F) values were measured in three spin-echo experiments conducted with relaxation delays of 8 μs, 40 ms and 80 ms. $R_{1\rho}$(^{19}F) values were measured in CPMG experiments with 0.4 ms spacing between subsequent 180° pulses. The relaxation delays used were 0.8, 7.2, 13.6, 20.0, 26.4, 32.8, 39.2, 45.6, 52.0, 58.4, 64.8, 71.2, 77.6 and 84.0 ms.

DFT calculations of ^{13}C chemical shifts and $^3J_{FC}$ coupling constants

Restricted Kohn-Sham density-functional theory (DFT) calculations were performed using the ωB97X-D3BJ functional (Najibi and Goerigk, 2018), D3BJ dispersion corrections (Grimme et al., 2010; 2011), the aug-cc-pvdz basis set (Kendall et al., 1992), and the RIJCOSX approximation for two-electron integrals (Neese, 2003; Neese et al., 2009; Helmich-Paris et al., 2021) with on-the-fly auxiliary basis sets (Stoychev et al., 2017), and in the presence of a solvent continuum (Garcia-Rates and Neese, 2020) with a dielectric constant of 6.5 and a refractive index of 1.4 in Orca 6.0.0 (Neese, 2022, 2023). Each valine residue and rotamer was taken from our previous work (Frkic et al., 2024). Calculations for the ^{13}C chemical shifts and $^3J_{FC}$ coupling constants were performed using the gauge-including atomic orbital (GIAO) method (Stoychev et al., 2018). To obtain the torsional dependence data, we optimised the structure of $(CH_3)_2CHCH_2F$ in the gas phase but including a solvent continuum (Garcia-Rates and Neese, 2020) with a dielectric constant of 6.5 and a refractive index of 1.4. The relative torsion of the methyl-C-C-C-F dihedral angle (i.e. a rotation of the -CH_2F group) was scanned without any other optimization and the ^{13}C chemical shifts and $^3J_{FC}$ coupling constants were calculated (Figure S7 and main text Figure 12b). We also performed another scan where the remaining degrees of freedom were relaxed during the torsion scan (Figure S8). The relaxed coordinates differ only marginally from the non-relaxed coordinates, but a notable change for $^3J_{FC}$ at $\tau = 0°$ was observed.

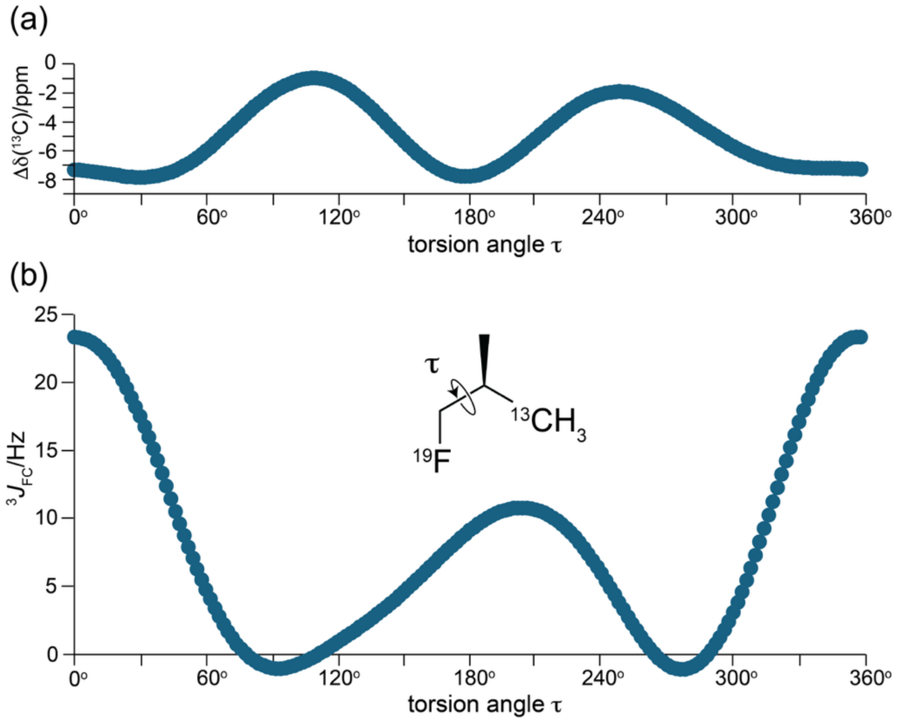

Figure S7. Torsion angle dependence of ^{13}C chemical shifts and $^{3}J_{FC}$ coupling constants predicted by DFT calculations for (2*R*)-1-fluoro-2-methylpropane(3-^{13}C). (a) $\Delta\delta(^{13}C)$ versus the torsion angle τ. The calculations were performed without relaxing the molecular geometry prior to calculating the NMR parameters. (b) $^{3}J_{FC}$ versus the torsion angle τ. See Figure 12 of the main text for a plot of $\Delta\delta(^{13}C)$ versus $^{3}J_{FC}$.

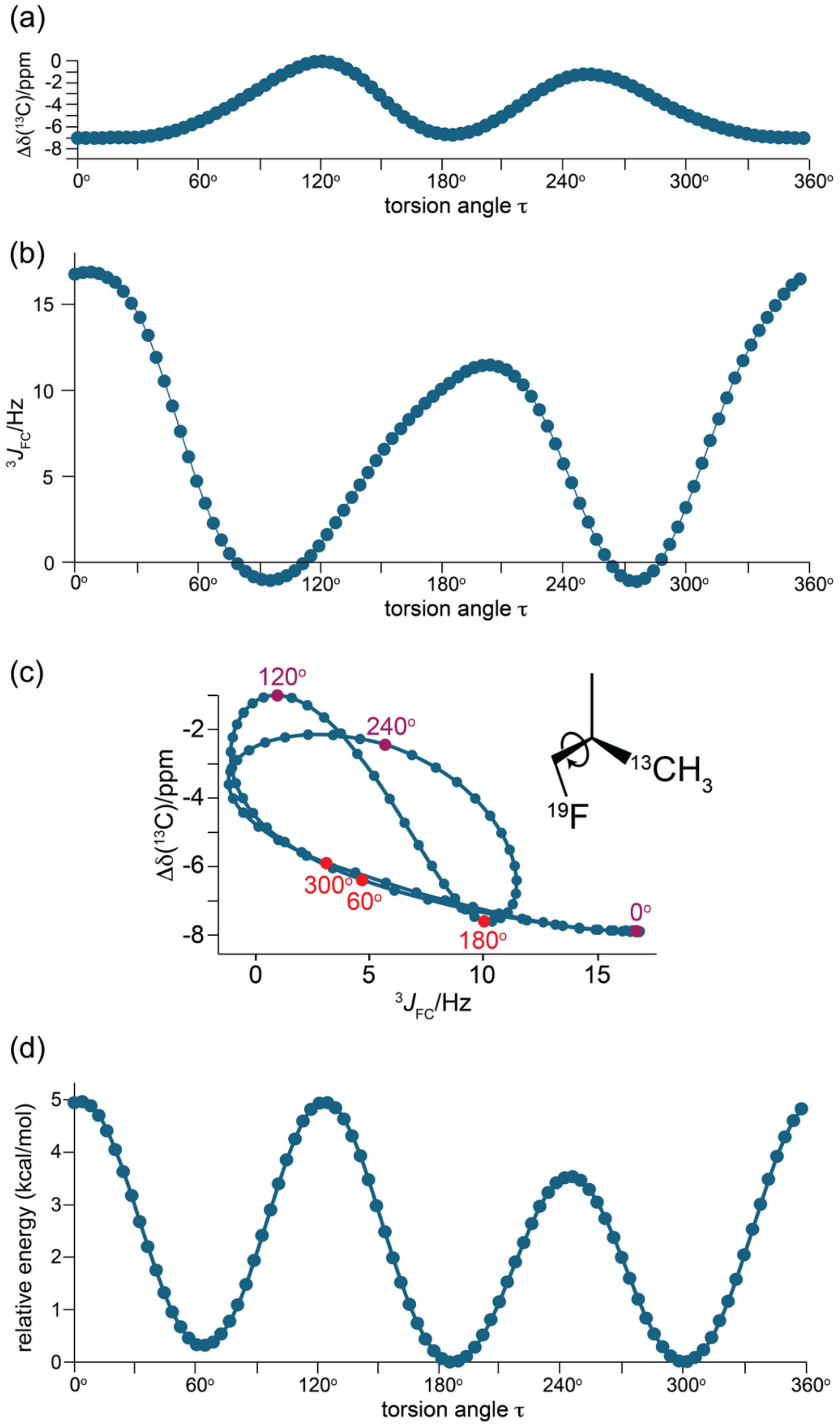

Figure S8. Calculated torsion angle dependence of ^{13}C chemical shifts and $^{3}J_{\mathrm{FC}}$ coupling constants in (2*R*)-1-fluoro-2-methylpropane(3-^{13}C) with relaxation of all other molecular degrees of freedom. (a) $\Delta\delta(^{13}\mathrm{C})$ versus the torsion angle τ. (b) $^{3}J_{\mathrm{FC}}$ versus the torsion angle τ. (c) Correlation of $\Delta\delta(^{13}\mathrm{C})$ with the $^{3}J_{\mathrm{FC}}$ coupling constants in the representation of Figure 12 of the main text. The points for the three staggered rotamers (±60° and 180°) are on a nearly straight line. (d) Calculated energies.

References

Frkic, R., Tan, Y. J., Maleckis, A., Chilton, N. F., Otting, G., and Jackson, C. J.: 1.3 Å crystal structure of *E. coli* peptidyl–prolyl isomerase B with uniform substitution of valine by (2*S*,3*S*)-4-fluorovaline reveals structure conservation and multiple staggered rotamers of CH_2F groups, Biochemistry, 63, 2602–2608, https://doi.org/10.1021/acs.biochem.4c00345, 2024.

Garcia-Rates, M. and Neese, F.: Effect of the solute cavity on the solvation energy and its derivatives within the framework of the Gaussian charge scheme, J. Comput. Chem., 41, 922–939, https://doi.org/10.1002/jcc.26139, 2020.

Grimme, S., Antony, J., Ehrlich, S., and Krieg, H.: A consistent and accurate ab initio parametrization of density functional dispersion correction (DFT-D) for the 94 elements H-Pu, J. Chem. Phys., 132, 154104, https://doi.org/10.1063/1.3382344, 2010.

Grimme, S., Ehrlich, S., and Goerigk, L.: Effect of the damping function in dispersion corrected density functional theory, J. Comput. Chem., 32, 1456–1465, https://doi.org/10.1002/jcc.21759, 2011.

Helmich-Paris, B., de Souza, B., Neese, F., and Izsák, R.: An improved chain of spheres for exchange algorithm. *J. Chem. Phys.*, *155*, 104109, https://doi.org/10.1063/5.0058766, 2021.

Juszewski, K., Gronenborn, A. M., and Clore, G. M.: Improving the packing and accuracy of NMR structures with a pseudopotential for the radius of gyration, J. Am. Chem. Soc., 121, 2337–2338, https://doi.org/10.1021/ja9843730, 1999.

Maleckis, A., Abdelkader, E. H., Herath, I. D., and Otting, G.: Synthesis of fluorinated leucines, valines and alanines for use in protein NMR, Org. Biomol. Chem., 20, 2424–2432, https://doi.org/10.1039/D2OB00145D, 2022.

Najibi, A. and Goerigk, L.: The nonlocal kernel in van der Waals density functionals as an additive correction: an extensive analysis with special emphasis on the B97M-V and ωB97M-V approaches, J. Chem. Theory Comput., 14, 5725–5738, https://doi.org/10.1021/acs.jctc.8b00842, 2018.

Neese, F.: An improvement of the resolution of the identity approximation for the formation of the Coulomb matrix, J. Comput. Chem., 24, 1740−1747, https://doi.org/10.1002/jcc.10318, 2003.

Neese, F.: Software update: the ORCA program system, version 5.0, WIRES Comput. Molec. Sci., 12, e1606, https://doi.org/10.1002/wcms.1606, 2022.

Neese, F.: The SHARK integral generation and digestion system, J. Comp. Chem., 44, 381–396, https://doi.org/10.1002/jcc.26942, 1−16, 2023.

Stoychev, G. L., Auer, A. A., and Neese, F.: Automatic generation of auxiliary basis sets, J. Theo. Comp. Chem., 13, 554–562, https://doi.org/acs.jctc.6b01041, 2017.

Stoychev, G. L., Auer, A. A., Izsak, R., and Neese, F.: Self-consistent field calculation of nuclear magnetic resonance chemical shielding constants using gauge-including atomic orbitals and approximate two-electron integrals, J. Chem. Theory Comput. 14, 619−637, https://doi.org/10.1021/acs.jctc.7b01006, 2018.

Tan, Y. J., Abdelkader, E. H., Herath, I. D., Maleckis, A., and Otting, G.: Through-space scalar ^{19}F–^{19}F couplings between CH_2F groups of leucine residues in a protein, Magn. Reson., 6, 131−142, https://doi.org/10.5194/mr-6-131-2025, 2025.
